# Hippocampal involvement in nonpathological déjà vu: Subfield vulnerability rather than temporal lobe epilepsy equivalent

**DOI:** 10.1002/brb3.1299

**Published:** 2019-05-15

**Authors:** Eva Pešlová, Radek Mareček, Daniel J. Shaw, Tomáš Kašpárek, Martin Pail, Milan Brázdil

This article (Pešlová et al., 2018) was also supported by the Ministry of Health of the Czech Republic, grant nr. 17‐32292A. All rights reserved.
